# Impact of O-Acetylation on *S. flexneri* 1b and 2a O-Antigen Immunogenicity in Mice

**DOI:** 10.3390/microorganisms9112360

**Published:** 2021-11-15

**Authors:** Vanessa Arato, Davide Oldrini, Luisa Massai, Gianmarco Gasperini, Francesca Necchi, Francesca Micoli

**Affiliations:** GSK Vaccines Institute for Global Health, 53100 Siena, Italy; vanessa.x.arato@gsk.com (V.A.); davide.x.oldrini@gsk.com (D.O.); luisa.x.massai@gsk.com (L.M.); gianmarco.x.gasperini@gsk.com (G.G.); francesca.x.necchi@gsk.com (F.N.)

**Keywords:** *Shigella flexneri*, O-antigen, O-acetylation, GMMA vaccine

## Abstract

Shigellosis is a diarrheal disease caused prevalently by *Shigella flexneri* and *S. sonnei* and representing a major global health risk, particularly in developing countries. Bacterial O-antigen (OAg) is the primary target of the host immune response and modifications of its oligosaccharide units, including O-acetylation, are responsible for the variability among the circulating *S. flexneri* serotypes. No vaccines are widely available against shigellosis and the understanding of the immunogenicity induced by the OAg is fundamental for the design of a vaccine that could cover the most prevalent *Shigella* serotypes. To understand whether a different O-acetylation pattern could influence the immune response elicited by *S. flexneri* OAg, we employed as a vaccine technology GMMA purified from *S. flexneri* 2a and 1b strains that were easily engineered to obtain differently O-acetylated OAg. Resulting GMMA were tested in mice, demonstrating not only no major impact of O-acetyl decorations on the immune response elicited by the two OAg against the homologous strains, but also that the O-acetylation of the Rhamnose III residue (O-factor 9), shared among serotypes 1b, 2a and 6, does not induce cross-reactive antibodies against these serotypes. This work contributes to the optimization of vaccine design against *Shigella*, providing indication about the ability of shared epitopes to elicit broad protection against *S. flexneri* serotypes and supporting the identification of critical quality attributes of OAg-based vaccines.

## 1. Introduction

*Shigella* infections are among the top causes of moderate to severe diarrhea throughout the world, causing approximately 112 million cases of which a great percentage is represented by children under 5 years of age [1].

There are four different *Shigella* species: *S. boydii*, *S. dysenteriae*, *S. flexneri*, and *S. sonnei*, divided in more than 50 serotypes based on antigenic variation of their O-Antigen (OAg). *S. flexneri* is predominant in low- and middle-income countries (LMIC) causing 66% of cases of shigellosis, with serotypes 1b, 2a, 3a, and 6 representing the most abundant ones [1,2,3]. The increasing emergence of multidrug-resistant clones makes *Shigella* even more worrisome, pressing the race for the development of a vaccine, still not widely available on the market [4,5,6,7]. However, several groups worldwide are making great efforts in testing candidate vaccines, most of them targeting the OAg portion of *Shigella* lipopolysaccharide (LPS) anchored through the lipid A to the outer membrane. LPS is the most relevant virulence factor in *Shigella* pathogenesis and plays an important role in bacterial resistance to both innate and adaptive immunity [8,9,10,11,12].

All *S. flexneri* serotypes, except *S. flexneri* 6, share a conserved polysaccharide backbone (also known as serotype Y) [13,14,15] consisting of the following repeating unit:

2)-α-L-Rha*p*III-(1,2)-α-L-Rha*p*II-(1,3)-α-L-Rha*p*I-(1,3)-β-D-Glc*p*NAc-(1-. 

**Figure 1:**
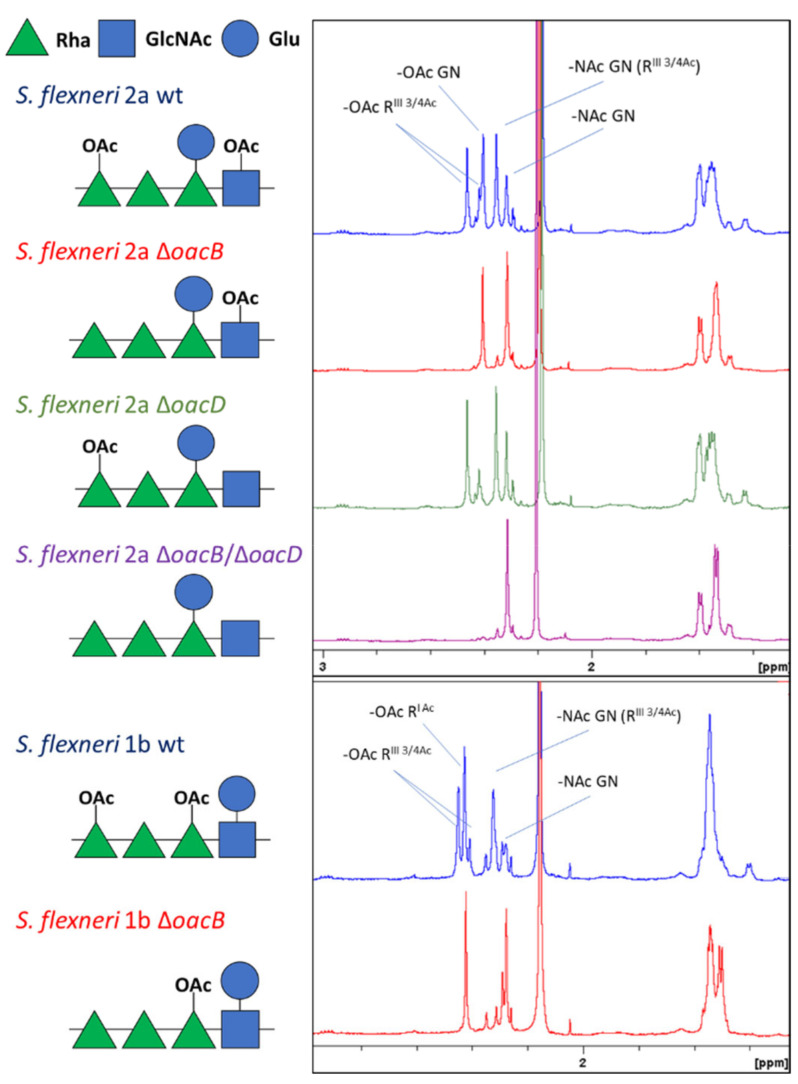
Schematic representation of differently O-acetylated *S. flexneri* 2a and 1b OAg structures generated in this study with the corresponding ^1^H NMR spectra focusing on the OAc area. R = Rha, GN = GlcNAc. Complete ^1^H NMR spectra are shown in Figure S1.

The diversity of the *S. flexneri* serotypes is due to the modification of the common OAg backbone with glucosyl and/or O-acetyl (OAc) groups as a result of bacteriophages infection and acquisition of OAg-modifying enzymes [16]. In the case of *S. flexneri* 2a OAg, the repeating unit is substituted with a glucose on RhaI, added by the glucosyltransferase GtrII (epitope shared with *S. flexneri* 2b) [17]. Moreover, two different O-acetyltransferases can modify the repeating unit by adding O-acetyl groups on GlcNAc (OacD) and/or on RhaIII (OacB) [18,19]. 6-O-acetylation on GlcNAc confers the antigenic group O-factor 10 [19]. *S. flexneri* 1b OAg repeating unit is substituted with a glucose on GlcNAc, added by the glucosyltransferase GtrI (epitope shared with *S. flexneri* 1a). The O-acetyltransferase OacA O-acetylates RhaI (epitope that allows discrimination with *S. flexneri* 1a), whereas the O-acetyltransferase OacB adds a 3/4 O-acetylation on RhaIII (Figure 1) [13,18]. The addition of an O-acetyl group on position 3 or 4 of RhaIII provides the antigenic group O-factor 9. 

Wang and colleagues studied the distribution of the 3/4-O-acetylation in *S. flexneri* and the antigenicity that resulted from this modification. PCR screening of the *oacB* gene in 730 clinical isolates of *S. flexneri* demonstrated that the *oacB*-mediated 3/4-O-acetylation is widespread in serotypes 1a, 1b, 2a, and Y. Among them, 1a, 1b, and 2a are the predominant serotypes in China and other Asian countries, and the high 3/4-O-acetylation rates (>94%) found in these serotypes suggest that the presence of O-factor 9 may play a role in their preferable dissemination. However further studies are necessary to elucidate if this OAg modification contributes to the virulence of *S. flexneri*. 

O-factor 9 has also been reported in *S. flexneri* serotype 6, despite its genetic determinant was found in the *oacC* gene (72% homologous to *oacB*) [20]. 

There are no direct evidence in the current literature about the role of O-acetylation in the immune response induced by *S. flexneri* OAg. It has been reported that a *S. flexneri* 2a OAg conjugate vaccine induced protection against *S. flexneri* serotype 6 in humans and the O-factor 9 was suggested as the structural basis for the observed cross-reactivity between *S. flexneri* 2a and *S. flexneri* 6 LPS [21,22].

In this study we aim to verify the criticality of O-acetylation pattern for *S. flexneri* OAg, in particular for *S. flexneri* 2a and 1b, two of the most prevalent *S. flexneri* serotypes, and the impact that OAc epitopes can have in the cross-recognition of homologous and heterologous *S. flexneri* serotypes. We used the generalized modules for membrane antigens (GMMA) technology to rapidly generate different OAg structures with the desired features. GMMA are outer membrane vesicles naturally released from genetically engineered Gram-negative bacteria, having the great advantage to display the OAg in its natural outer membrane context. *Shigella* bacteria are mutated in order to increase vesicles formation, through the deletion of the *tolR* gene [23]. Potential reactogenicity is reduced by modifying the lipid A structure usually by deletion of the *htrB* or *msbB* genes [24]. For these advantages and their easy and cost-effective manufacturability, GMMA have been proposed as a strategy for the development of a multi-component vaccine against *Shigella* [25,26,27,28,29]. Starting from overblebbing Δ*tolR S. flexneri* 1b and 2a strains, we generated knock-out mutants of *S. flexneri* 2a for the O-acetyltransferases genes *oacB* and *oacD* and of *S. flexneri* 1b for the O-acetyltransferase gene *oacB*. GMMA obtained from these strains were used for immunogenicity studies in mice. Notably, OAc on RhaI of *S. flexneri* 1b was not altered as its removal would convert the strain to serotype 1a.

The results from this study will contribute to the design of cross-protective OAg-based vaccines against *Shigella flexneri*.

## 2. Materials and Methods

### 2.1. Bacterial Strains and Generation of Mutants

*S. flexneri* 2a and *S. flexneri* 1b were acquired from Public Health England (PHE) and engineered to obtain the different mutants. The null mutations were obtained by replacing the genes of interest with an antibiotic resistance cassette by homologous recombination using the lambda red recombineering system [30]. The list of all bacterial strains generated and primers used is reported in Supplementary Table S1. The O-acetyltransferases null mutations were inserted both in a wild type and Δ*tolR* background to generate bacterial strains to be used for serum bactericidal assay (SBA) and GMMA purification, respectively. Absence of O-acetyltransferase genes was confirmed by PCR and the phenotype of each mutant was verified by H^1^ NMR analysis of OAg extracted from the resulting GMMA, as previously described [31,32]. 

### 2.2. GMMA Production and Characterization

*S. flexneri* 2a and 1b GMMA were produced and purified as previously described [31]. GMMA total protein content was estimated by bicinchoninic acid assay (BCA) using BSA as a reference. The total OAg amount and sugar composition were determined by high-performance anion-exchange chromatography with pulsed amperometric detection (HPAEC–PAD), after performing acid hydrolysis directly on GMMA. In particular, the OAg amount was quantified based on the detection of rhamnose, as previously described [33,34]. The OAg extracted was characterized by high performance liquid chromatography–size exclusion chromatography (HPLC-SEC) (TSK gel 3000 PWXL column with TSK gel PWXL guard column equilibrated in 0.1 NaCl, 0.1 NaH_2_PO_4_, 5% CH_3_CN, Tosoh Bioscience, Tokyo, Japan) with differential refractive index (dRI) detection, using dextrans as the standards to estimate the molecular size distribution as previously described [31,33]. OAg structures were confirmed by ^1^H-NMR analysis measured with a Bruker AvanceIII 400 spectrometer at 400 MHz and 323 K.

### 2.3. Mouse Studies 

GMMA obtained from *S. flexneri* 2a and 1b, differing for O-acetylation pattern, were tested in mice. Female, five-week-old wild-type CD1 mice were immunized intraperitoneally (IP) with 200 µL of vaccine containing 0.005, 0.05, or 0.5 µg of OAg at day 0 and 28. Single sera were collected at day 27 and 42. All animal studies were performed at GSK ARC Animal Care Facility under the animal project 526/2020-PR 26/05/2020 approved by the Italian Ministry of Health. The studies were ethically reviewed and carried out in accordance with European Directive 2010/63/EEC, the GSK policy on the Care, Welfare and Treatment of Animals, and local animal welfare guidelines under Italian authorization. All sera were analyzed by ELISA for anti-*S. flexneri* OAg total IgG using wild type *S. flexneri* 1b and 2a OAg as plate-coating antigens (at 0.5 and 5 µg/mL concentration in carbonate buffer pH 9, respectively). All coating antigens were purified in house as previously described [33]. Single sera collected at day 42 were also assayed in SBA based on luminescent readout (L-SBA) [35] against *S. flexneri* 1a, 1b, 2a, 2b, or 6 wild-type or mutant strains, as previously described [28,31].

### 2.4. Statistical Analysis

Analysis was performed using GraphPad Prism 7 (La Jolla, CA, USA). The Kruskal–Wallis analysis with post-hoc Dunn’s test was used to compare multiple groups. Dose–response relationships were evaluated through Spearman’s rank correlation. The parallelism of dose–response curves was assessed by the parallel line method: when the slopes of the curves obtained by log-transforming ELISA or SBA results vs. log transformed antigen doses were not significantly different from each other, comparison of the Y-intercepts was performed.

## 3. Results

### 3.1. Generation of Differently O-Acetylated S. flexneri 1b and 2a Strains and Characterization of the Resulting GMMA

*S. flexneri* 1b and 2a strains were genetically manipulated to obtain overblebbling phenotypes through knock-out of the *tolR* gene. With the aim to verify the impact of OAg O-acetylation on the immune response induced in mice, genes coding for O-acetyltransferases were also removed and GMMA producing strains differing for the OAg O-acetylation pattern were obtained, as shown in Figure 1.

GMMA were purified from each *S. flexneri* strain and analytically characterized (Table 1). Importantly, mutations introduced did not impact the OAg amount in *S. flexneri* 2a GMMA as all GMMA were characterized by similar OAg to protein ratios, estimated through HPAEC-PAD and micro BCA analyses. GMMA displaying a mutated OAg for *S. flexneri* 1b presented instead a slightly decreased OAg/protein ratio with respect to its wild-type counterpart.

The OAg was extracted from each GMMA and analyzed through HPLC-SEC to determine the OAg molecular weight (MW) distribution. Importantly, introduced mutations did not impact OAg size. Indeed, the four strains of *S. flexneri* 2a showed very similar percentage of three different OAg populations: around 15% for the high MW (HMW), around 50% for the medium MW (MMW), and around 35% for the low MW (LMW) OAg (this peak corresponds to core only and core plus few OAg repeats). Moreover, the two *S. flexneri* 1b strains resulted to have very similar OAg populations: 65% and 35% for the MMW and LMW, respectively.

The phenotypes resulting from the genetic deletion of the O-acetyltransferase genes were confirmed through ^1^H NMR analysis. *S. flexneri* 2a wild type (wt) OAg was 71% O-acetylated on RhaIII (position 3/4) and 56% O-acetylated on GlcNAc. *S. flexneri* 2a OAg from strains lacking *oacB* or *oacD* were 65% and 66% O-acetylated on GlcNAc and RhaIII, respectively. As expected, no signals related to OAg OAc were detected in the ^1^H NMR spectrum for the double knock-out mutant Δ*oacB*/Δ*oacD* of *S. flexneri* 2a, confirming complete OAg de-O-acetylation. *S. flexneri* 1b wt OAg was 63% O-acetylated on RhaI and 80% O-acetylated on RhaIII. The OAg from the strain lacking *oacB* was 79% O-acetylated on RhaI and not O-acetylated on RhaIII, as expected (Figure 1 and Figure S1). Importantly, deletion of OAc groups in specific positions did not impact the O-acetylation degree in the remaining ones, allowing a good comparison among the different constructs in mice.

### 3.2. Immunogenicity Study in Mice with OAg Differing for the OAc Pattern

All GMMA were compared in mice at three different OAg doses (0.5, 0.05, and 0.005 µg) and mice were immunized intraperitoneally twice at 4-week intervals.

**Figure 2:**
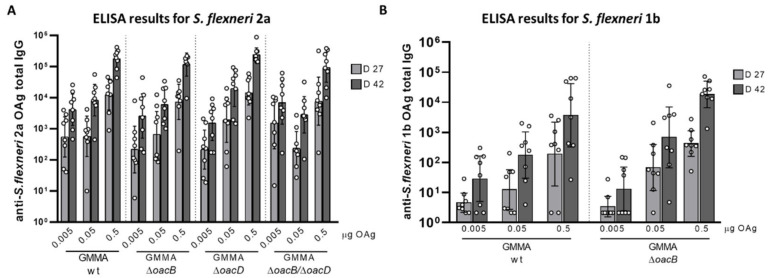
ELISA results of total anti-OAg specific IgG of *S. flexneri* 2a GMMA (**A**) and *S. flexneri* 1b GMMA (**B**) differing for OAg O-acetylation. Eight wild type (CD1) mice per group were intraperitoneally immunized on days 0 and 28, with 0.005, 0.05, and 0.5 µg OAg dose. Summary graphs of anti-OAg specific IgG report individual antibody levels (dots) and geometric mean with 95% confidence interval (bars).

Mice sera obtained after first (day 27) and second immunization (day 42) were analyzed through ELISA using *Shigella* wt fully O-acetylated OAg as coating antigen for each *S. flexneri* serotype. For both *S. flexneri* 2a and 1b, the anti-OAg IgG response induced in mice was not impacted by the OAg O-acetylation pattern, as the anti-OAg IgG obtained by immunizing with the differently O-acetylated GMMA was not statistically different neither at day 27 nor at day 42 (Figure 2). These data were analyzed by applying the parallel line approach, which revealed no significant differences between wild type and mutated GMMA in the range of OAg doses investigated.

**Figure 3:**
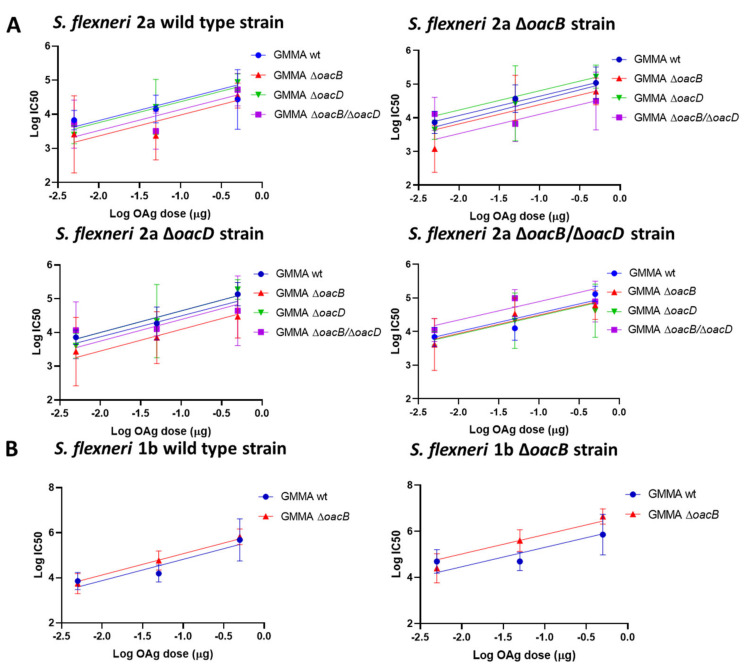
L-SBA results against homologous strains differing for O-acetylation pattern (indicated as titles of each graph). Parallel line analysis showed that slopes and Y-intercepts of the dose-response curves of the different formulations (reporting log transformed IC50 titers in Y axis and log transformed OAg doses in X axis) were not statistically significantly different both in the case of *S. flexneri 2a* (**A**) and *S. flexneri* 1b (**B**), being their *p*-values above the specified alpha of 0.05.

Sera collected at day 42 were further analyzed for antibodies functionality by L-SBA performed against each of the corresponding differently O-acetylated *Shigella* strain. L-SBA data confirmed the results obtained by ELISA and proved that the functional activity of sera increased against each GMMA was comparable regardless the presence of OAc groups (Figure 3). The different GMMA groups were compared to each other by applying the parallel line approach, which revealed no significant differences in the slopes and Y-intercepts as shown in Figure 3.

To understand weather O-acetylation can impact the ability of *S. flexneri* OAg to raise cross-functional antibodies against heterologous serotypes, sera obtained by immunizing mice with the highest *S. flexneri* 2a and 1b OAg dose were tested in L-SBA also against *S. flexneri* 1b and 2a, respectively as well as against other heterologous *S. flexneri* serotypes. 

In particular, for what concerns *S. flexneri* 2a, besides *S. flexneri* 1b, we selected *S. flexneri* serotypes 1a and 6 that also are O-acetylated on RhaIII and serotype 2b that shares with *S. flexneri* 2a the glucosylation on RhaI (epitope conferring the type 2 serotype). 

**Figure 4:**
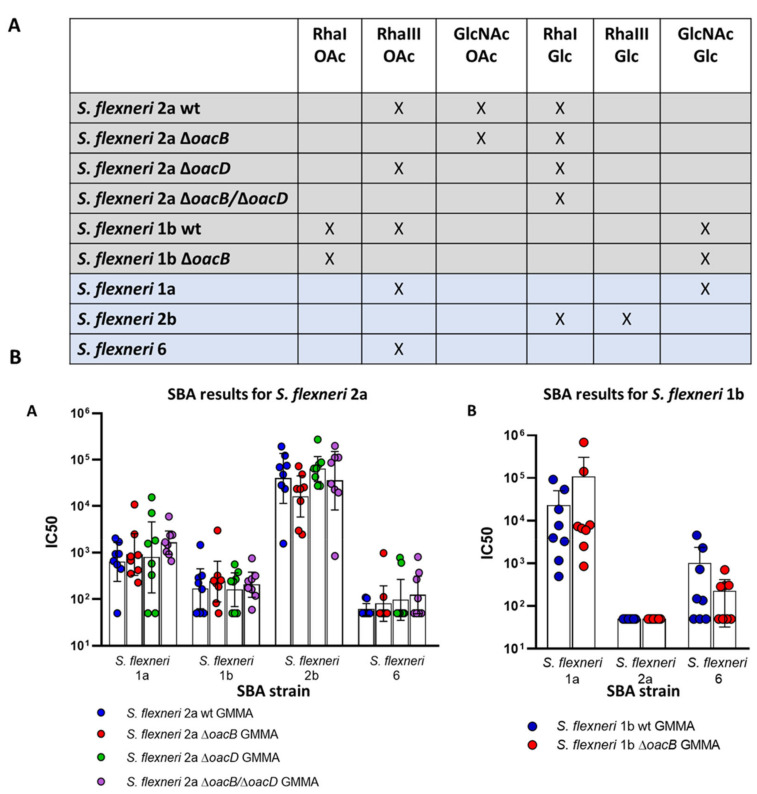
SBA results against heterologous strains. (**A**) Table showing OAg epitopes shared among the *Shigella* serotypes used in this study, including GMMA producing strains of *S. flexneri* 2a and 1b (grey rows) and *S. flexneri* serotypes used to check cross-reactivity in SBA (blue rows). (**B**). Summary graphs of L-SBA titers against the heterologous *S. flexneri* strains (X axis) reporting individual IC_50_ levels (dots) and geometric mean (bars). Sera tested in this assay were raised by immunizing mice with 0.5 µg of GMMA (OAg dose) from *S. flexneri* 2a (**B-A**) and *S. flexneri* 1b (**B-B**). Statistics performed by applying a Kruskal–Wallis test among the differently O-acetylated GMMA is not reported as it resulted in non-significant differences for all the strains tested.

SBA results, reported in Figure 4, clearly show that all sera raised against *S. flexneri* 2a GMMA resulted in high bactericidal titers against *S. flexneri* 1a and 2b (IC_50_ 10^3^–10^5^), while relatively low titers were measured against *S. flexneri* 1b and 6 (IC_50_ < 10^3^). However, the presence/absence of OAc on *S. flexneri* 2a OAg did not make any difference in the functionality of the antibodies raised, indicating that common OAg OAc did not play a role in inducing cross-reactive antibodies across the heterologous *S. flexneri* strains tested. 

Similarly, polyclonal sera obtained with *S. flexneri* 1b GMMA were tested in SBA against *S. flexneri* 1a, 2a, and 6 that also present OAc on RhaIII. Serum bactericidal killing of *S. flexneri* 1a was high (IC_50_~10^5^), whereas low IC_50_ titers were measured when sera were tested against *S. flexneri* 6 and almost no killing was observed in the case of *S. flexneri* 2a. These results were independent from the presence of O-acetylation on *S. flexneri* 1b OAg.

## 4. Discussion

*Shigella* is a major cause of morbidity worldwide and antimicrobial resistance makes the development of a widely accessible vaccine an even higher global health priority [4]. The major challenge for the development of a vaccine against *Shigella* is related to the high number of serotypes causing the disease. In response to this need, multi-component vaccines are currently under development to ensure broad protection against *Shigella* [36,37].

It is known that structural characteristics of saccharide antigens, including O-acetylation, can play a major role on the immune response elicited and can be critical for the design of highly effective vaccines [38]. This is the case of *Salmonella* Typhi and S*almonella* Paratyphi A, where the Vi and OAg immunogenicity were related to their degree of O-acetylation [39,40]. On the other hand, polysaccharide O-acetylation was not found to be essential for antigenicity and immunogenicity of some *Streptococcus pneumoniae* strains [41], however it is an aspect not to underestimate in the context of vaccine development.

Few preclinical data have been collected so far on the role that O-acetylation can have on the immune response elicited by *S. flexneri* OAg. This can be important not only to assess the protective role against homologous strains, but also against heterologous serotypes sharing common epitopes due to such modifications. Indeed, cross-reactivity could help to reduce the complexity of a *Shigella* vaccine composition. 

GMMA represent a powerful technology to facilitate this kind of investigation, besides being a promising vaccine approach [42]. Indeed, GMMA-producing strains can be easily mutated to display OAg with the desired features and resulting GMMA can be rapidly tested in animal studies. Here we have generated GMMA displaying *S. flexneri* 2a and 1b OAg with different O-acetylation patterns by knocking-out the responsible genes, similarly to what has already been reported to obtain different OAg size [43].

The impact that OAg O-acetylation has on several aspects of the *Shigella* pathogenesis and immunogenicity has never been clarified so far. In this study we looked at the role of *S. flexneri* 2a and 1b OAg O-acetylation in driving an immune response in mice, when delivered with GMMA. In particular, the aim was to verify the criticality of O-acetylation occurring in two positions of *S. flexneri* 2a OAg, on RhaIII and on GlcNAc, and on RhaIII of *S. flexneri* 1b in generating a broad and functional anti-OAg immune response. 

The data obtained suggested that the OAc on RhaIII or/and GlcNAc of *S. flexneri* 2a OAg and the OAc on RhaIII of *S. flexneri* 1b OAg are not critical for the generation of functional antibodies against homologous or heterologous strains.

These results are in line with a recent publication in which we showed that *S. flexneri* 6 OAg O-acetylation has little effect on OAg conformation and hence may not be essential for the antigenicity of serotype 6. This was corroborated by a similar in vivo study in mice, using *S. flexneri* 6 GMMA as O-antigen delivery systems, that shows that O-acetylation does not have an impact on the immune response elicited by the serotype 6 O-antigen [32]. Moreover, binding studies of synthetic *S. flexneri* 2a OAg to five protective mIgGs showed that none of the acetates affect significantly the antibody recognition [44]. However, it is fair to highlight that conclusions drawn on studies done using the mouse model may not reflect the effects of OAg O-acetylation in immune response occurring in humans. Indeed, as previously mentioned, a *S. flexneri* 2a conjugate vaccine was able to elicit in humans cross-reactive antibodies against *S. flexneri* 6 and is reasonable to attribute this result to the O-factor 9 (OAc of RhaIII), the only epitope that these two OAg structures have in common, although no cross-reactivity was observed from studies done in the mouse model, as also confirmed by our data, independently from the O-acetylation pattern [21]. Moreover, Hlozek et al. recently performed computational investigation of the effect of O-acetylation of RhaIII in serogroup 2 on OAg conformation, showing that this modification limits the flexibility of the OAg backbone, changing the dominant conformation from a C-curve to a helix [45]. This change was suggested to provide a rationale for the cross-reactivity observed between antibody raised by an O-acetylated 2a vaccine and serotype 6. Finally, it is worth to remind that in the context of cross-reactivity among different *Shigella* serotypes, besides O-acetylation, also OAg glucosylation may play a role in generating conformational epitopes shared among different OAg structures and this aspect deserves further investigation.

Not necessarily data in mice will be predictive of data in humans, but the systematic study performed here allowed to collect a certain data package that will be corroborated from data from clinical trials soon. Different *Shigella* OAg-based vaccines are being tested in clinical trials and analysis of the sera for their bactericidal activity against a panel of different serotypes will allow to confirm or disprove preclinical data and help also clarifying the role of O-acetylation in the immune response elicited by *Shigella* OAg.

## Figures and Tables

**Table 1:** Analytical characterization of GMMA displaying OAg with different O-acetylation.

GMMA	Mutation	OAg/Proteins *w*/*w* Ratio	OAg OAc	OAg MW
*S. flexneri* 2a	Δ*tolR*	0.61	71% RhaIII 56% GlcNAc	15% HMW (59.4 kDa) 50% MMW (13.7 kDa) 35% LMW (1.8 kDa)
	Δ*tolR* Δ*oacB*	0.62	65% GlcNAc	
	Δ*tolR* Δ*oacD*	0.65	66% RhaIII	
	Δ*tolR* Δ*oacB* Δ*oacD*	0.61	0	
*S. flexneri* 1b	Δ*tolR*	0.84	63% RhaI 80% RhaIII	65% MMW (13.8 kDa) 35% LMW (1.7 kDa)
	Δ*tolR* Δ*oacB*	0.67	79% RhaI	

## Data Availability

Not applicable.

## Supplementary Materials

The following are available online at https://www.mdpi.com/article/10.3390/microorganisms9112360/s1, Figure S1: Complete ^1^H NMR spectra of OAg structures of the differently O-acetylated *S. flexneri* strains., Table S1:List of bacterial strains and primers used in this study.
