# Supplementary material for: Impact of O-Acetylation on S. flexneri 1b and 2a O-Antigen Immunogenicity in Mice
Source: Microorganisms. 2021 Nov 15;9(11):2360. doi: 10.3390/microorganisms9112360 (PMC8623282; doi:10.3390/microorganisms9112360)
Supplement: Supplementary file 1 [file microorganisms-09-02360-s001.zip › microorganisms-1432174-supplementary.pdf]

microorganisms

MDPI

Article

Impact of O-Acetylation on *S. flexneri* 1b and 2a O-antigen Immunogenicity in Mice

Vanessa Arato [1], Davide Oldrini[1], Luisa Massai [1], Gianmarco Gasperini [1], Francesca Necchi [1], Francesca Micoli [1]

[1] GSK Vaccines Institute for Global Health.
* Correspondence: francesca.x.micoli@gsk.com

Supplementary figures and tables

Figure S1. Complete ^{1}H NMR spectra of OAg structures of the differently O-acetylated *S. flexneri* 2a (A) and 1b (B) strains generated in this study. R=Rha, GN=GlcNAc.

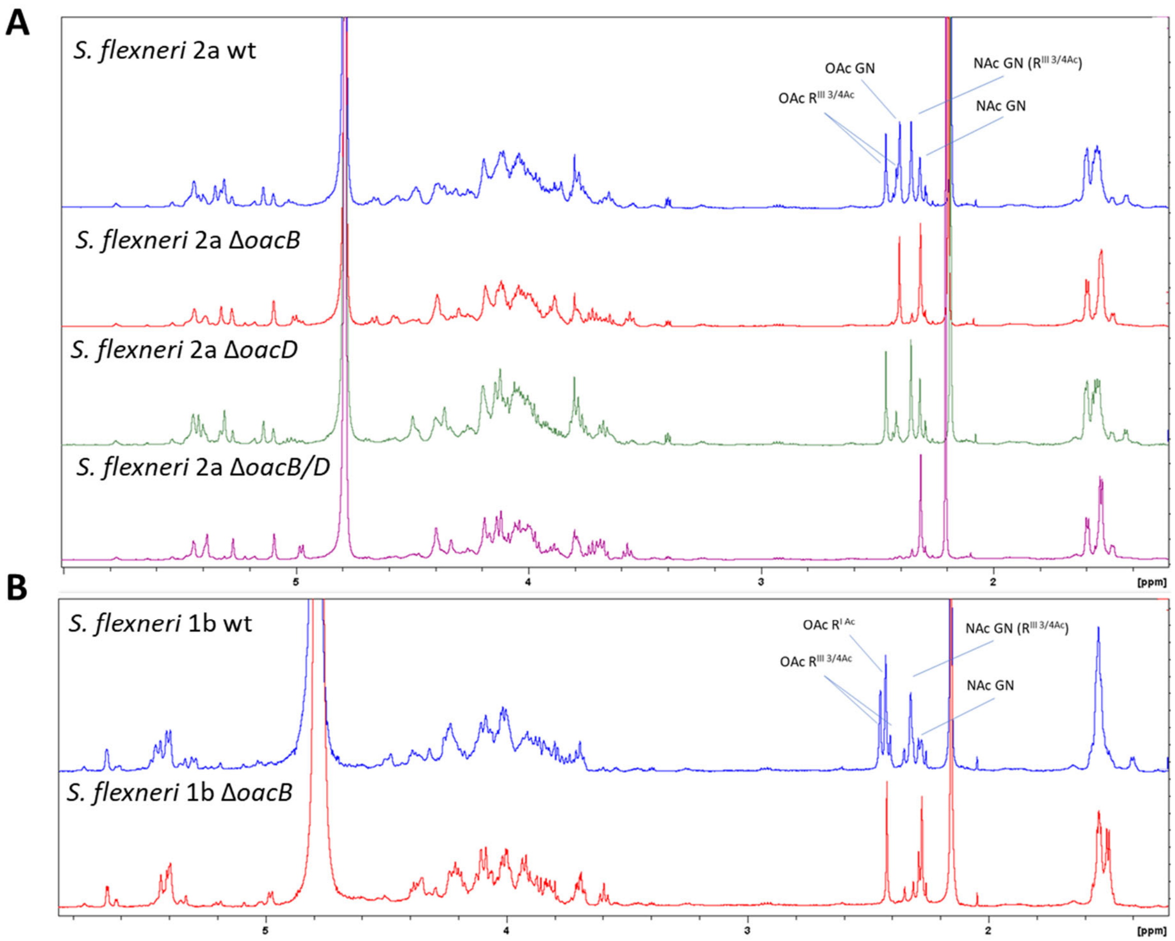

microorganisms

MDPI

Table S1. List of bacterial strains and primers used in this study.

Bacterial strains		
	**Name**	**Genotype**
**GMMA-producing strains**	*S. flexneri* 2a wt	*S. flexneri* 2a PHE-H130920142 *tolR*
	S. flexneri 2a Δ*oacB*	*S. flexneri* 2a PHE-H130920142 *tolR* Δ*oacB::aph*
	S. flexneri 2a Δ*oacD*	*S. flexneri* 2a PHE-H130920142 *tolR* Δ*oacD::cat*
	S. flexneri 2a Δ*oacB*/ Δ*oacD*	*S. flexneri* 2a PHE-H130920142 Δ*tolR* Δ*oacD::aph* Δ*oacB::cat*
	S. flexneri 1b wt	*S. flexneri* 1b PHE- H130920140 Δ*tolR*
	S. flexneri 1b Δ*oacB*	*S. flexneri* 1b PHE- H130920140 Δ*tolR* Δ*oacB::aph*
**SBA strains**	*S. flexneri* 2a wt	*S. flexneri* 2a 2457A
	S. flexneri 2a Δ*oacB*	*S. flexneri* 2a PHE-H130920142 Δ*oacB::cat*
	S. flexneri 2a Δ*oacD*	*S. flexneri* 2a PHE-H130920142 Δ*oacD::cat*
	S. flexneri 2a Δ*oacB*/ Δ*oacD*	*S. flexneri* 2a PHE-H130920142 Δ*oacD::aph* Δ*oacB::cat*
	S. flexneri 1b wt	*S. flexneri* 1b PHE-H130920140
	S. flexneri 1b Δ*oacB*	*S. flexneri* 1b PHE-H130920140 Δ*oacB::aph*
	S. flexneri 1a	*S. flexneri* 1a PHE-H130920139
	S. flexneri 2b	*S. flexneri* 2b PHE-H130920142
	S. flexneri 6	*S. flexneri* 6 PHE-H130920152

Primers	
**Name**	**5′-3′ sequence**
OacB KO Fwd (*aph/frt*)	TGCAATGAGTGTTTAAAAATCACACAAAACTGGAGAG CCTACTTTAAATGGTGTAGGCTGGAGCTGCTTC
OacB KO Rev (*aph/frt*)	TTTTATCAAAAATGGCCCACCGACTGTCCTCGATAGCC AATGGCCAATTTCATATGAATATCCTCCTTAG
OacB KO Fwd (*cat/frt*)	TGCAATGAGTGTTTAAAAATCACACAAAACTGGAGAG CCTACTTTAAATGGTGTAGGCTGGAGCTGCTTC
OacB KO Rev (*cat/frt*)	TTTTATCAAAAATGGCCCACCGACTGTCCTCGATAGCC AATGGCCAATTTCATATGAATATCCTCCTTAG
OacD KO Fwd (*cat/frt*)	TGCAATGAGTGTTTAAAAATCACACAAAACTGGAGAG CCTACTTTAAATGGTGTAGGCTGGAGCTGCTTC
OacD KO Rev (*cat/frt*)	TTTTATCAAAAATGGCCCACCGACTGTCCTCGATAGCC AATGGCCAATTTCATATGAATATCCTCCTTAG
tolR KO Fwd (*aph/frt*)	ACCGCCAGGCGTTTACCGTTAGCGAGAGCAACAAGGG GTAAGCCATGGCCGTGTAGGCTGGAGCTGCTTC
tolR KO Rev (*aph/frt*)	ACCCGCTCTCTTTCAAGCAAGGGAAACGCAGATGTTTA GATAGGCTGCGTCATATGAATATCCTCCTTAG

microorganisms

MDPI
